# Numerical Assessment of the Risk of Abnormal Endothelialization for Diverter Devices: Clinical Data Driven Numerical Study

**DOI:** 10.3390/jpm12040652

**Published:** 2022-04-18

**Authors:** Denis Tikhvinskii, Julia Kuianova, Dmitrii Kislitsin, Kirill Orlov, Anton Gorbatykh, Daniil Parshin

**Affiliations:** 1 Lavrentyev Institute of Hydrodynamics SB RAS, Lavrentiev Avenue 15, 630090 Novosibirsk, Russia; d.tikhvinskii@g.nsu.ru (D.T.); i.kuianova@g.nsu.ru (J.K.); 2 Neurosurgery Department, Meshalkin National Medical Research Center, 630055 Novosibirsk, Russia; kislitsinmd@gmail.com (D.K.); orlov72@mail.ru (K.O.); antonosjn@mail.ru (A.G.)

**Keywords:** cerebral aneurysm, flow diverting device, occlusion, artery stenosis, wall shear, endothelialization, computational fluid dynamics

## Abstract

Numerical modeling is an effective tool for preoperative planning. The present work is devoted to a retrospective analysis of neurosurgical treatments for the occlusion of cerebral aneurysms using flow-diverters and hemodynamic factors affecting stent endothelization. Several different geometric approaches have been considered for virtual flow-diverters deployment. A comparative analysis of hemodynamic parameters as a result of computational modeling has been carried out basing on the four clinical cases: one successful treatment, one with no occlusion and two with in stent stenosis. For the first time, a quantitative assessment of both: the limiting magnitude of shear stresses that are necessary for the occurrence of in stent stenosis (Max*WSS* > 1.23) and for conditions in which endothelialization is insufficiently active and occlusion of the cervical part of the aneurysm does not occur (Max*WSS* < 1.68)—has been statistacally proven (*p* < 0.01).

## 1. Introduction

Cerebral aneurysm (CA) is a common vascular disease which occurs in an average of 20–50 people per 1000 population [1,2]. Genetic and morphological aneurysm characteristics are the main parameters for studying when CA rupture risk is of interest [3]. Also hemodynamic parameters and sometimes a combination of hemodynamic and morphological parameters can predict aneurysm rupture [4]. However in spite of existence of many papers in this area a common solution to rupture risk assessment is not still clear and the main tasks of modern neurosurgery of CA are to determine the risk of their rupture [5,6] and recanalization [7,8]. Many studies are devoted specifically to the risk of rupture assessment for CA, while much less attention is paid to assessing their recanalization and finding the causes of abnormal endothelialization of flow-diverting devices particularly. The technique of flow-diverter device (FDD) placement in the last decade has been proven as a reliable method of aneurysm occlusion [9,10]. Meanwhile, up to 10% of all endovascular [11] interventions require reoperation due to abnormal endothelialization of FDD.

In the literature, there are quite a few works devoted to numerical [12,13,14,15,16], laboratory [17] modeling and clinical studies [18,19,20,21,22,23] of the installation of FDDs in cerebral vessels. However, most of these works give only the results of changes in hemodynamic characteristics [24], without linking them to a clinical application, and in some cases concern idealized models [25].

The development of an aneurysm is influenced by many factors, but major of them are: biochemical (such as the secretion of matrix metalloproteinase types 2 and 9, the secretion of growth factors due to intraluminal thrombosis); and biomechanical (such as wall shear stress and a relative residence time) [26]. The interrelation of morphological and hemodynamic characteristics as well as inflammation triggers provoke and enhance the remodeling of the aneurysm wall, which leads to an increase in its size and excretion of the wall of the aneurysmal sac [27].

In previous studies, *WSS* was known as one of the most important hemodynamic factors in aneurysm formation due to the relationship between high *WSS* and endothelial cells [28,29].

Retrospective virtual placement of a stent in the cerebral artery has been repeatedly modeled in the literature. There are several approaches to the modeling of FDD: modeling a detailed structure [30,31,32,33] and representing it as a region with a porous medium [34,35]. In our work, we use the second approach, when the volume occupied by the stent in the vessel is described by the equations of a porous medium. At the same time, we considered various options for the shape of the stent, which is associated with the differences in devices used in medical practice and the way they are installed.

The aim of this work is to study the hemodynamic reasons of recanalization of cerebral aneurysms during their treatment by installing FDDs, as well as to find out the hemodynamic factors of abnormal stent endothelialization. It turned out that absolute and relative values of changing of *WSS* values (before and after virtual stent deployment) in stent region is able to devide a sample into cohorts with respect to their clinical outcome.

## 2. Materials and Methods

In this paper, we retrospectively consider the cases of four patients Table 1 in whom the treatment of aneurysm was performed using the installation of FDDs. For the four patients, 3 approaches were considered.

**Figure 1:**
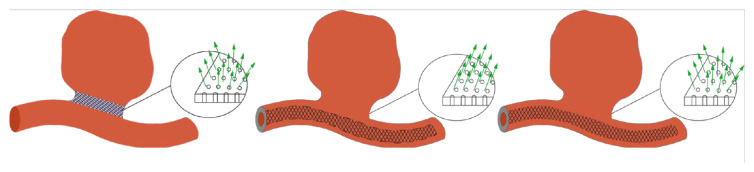
Schematic representation of approaches to stent deployment in Patients. (on the **left**) scenario A: the stent is a porous plug in the neck of the aneurysm; (in the **center**) scenario B: the stent is a porous tube along the vessel wall (the model is not isotropic); (on the **right**) scenario C: the stent is a porous tube along the vessel wall (isotropic model).

For virtual stent placement in a patient-specific geometry, which was obtained by using DICOM reconstruction of 3D angiography [36] of these patients with cerebral aneurysms. In all the considered cases, the area occupied by the stent is considered as a porous medium, but the geometry of the stents is different (Figure 1):

- Scenario A. The stent appears to be maximally apposed inside the vessel in such a way that its struds are pressed into the walls of the artery. In this setting, the stent interacts with the blood flow only at the orifice of the aneurysm. Thus, in this scenario, the stent looks like a layer of porous medium between the vessel body and the dome of the aneurysm;
- Scenario B. The stent is partially pressed into the vessel wall, it looks like a tube running along the center line of the vessel with a thickness of 0.8 mm [37] for patient 2 and 0.5 mm for patient 4. In this case, the tensor that determines the direction of the energy dissipation of the blood flow passing through the stent area is non-isotropic. This means that the direction is marked along the normal vector to the tube surface inside the aneurysm. This setting adequately simulates a small cell stent;
- Scenario C. This scenario is similar to scenario B without choosing the direction of energy dissipation. In this case, the tensor, which determines the direction of dissipation of the energy of the blood flow passing through the area of the stent, is isotropic. This setting corresponds to the wide-mesh stent model.
- Scenario 3.

It should be noted that all the scenarios under consideration are of clinical significance, since the location of the stent in the vessel is determined not only by the size of an aneurysm, but also by the geometry of the maternal vessel, as well as by the presence of perforator orifices at the stent-covered area.

### 2.1. Receiving the Data

CT imaging and surgical treatment were acquired at the Meshalkin National Medical Research Center of Ministry of Healthcare of The Russian Federation. The study was conducted in accordance with local ethic committee guidelines, all patients gave their informational consent and all patient data was anonymized prior to analysis.

### 2.2. Reconstruction of Blood Vessels

**Figure 2:**
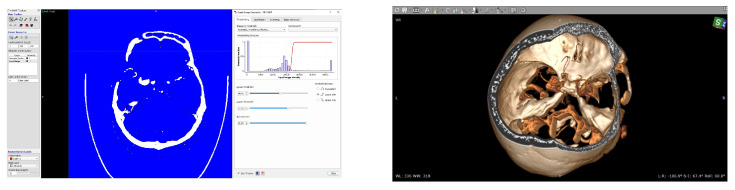
Vascular reconstruction methods: Image processing in the ITK-Snap program: DICOM image of the slice before the operation (**left**), Determination of the intensity threshold (**right**), Reconstruction of blood vessels in RadiAnt DICOM Viewer.

The process of the restoring geometry before and after the operation is as follows. The data array with the specified parameters was processed in an open access program for the segmentation of medical 3D images ITK-Snap [38] and commercial software Radiant DICOM Viewer (Poland, license of LIH SB RAS). These two programs allow t perform a segmentation of different quality and robustess level and have their own andvantages and disadventages. ITK Snap is more powerful to make the segmentation process more robust meanwhile Radiant is more easy, fast and native in use even for neurosurgeons. For the purposes of the current study no significant differences were found between the results of segmentation. Tomography data represent the dependence of the brightness function A(x,y,x)=F(v(x,y,z),n), where (x,y,z) is a point on the cut, v(x,y,z) is the blood flow velocity vector and n is the normal to the scanning plane. The problem of vasculature restoration is to define the boundaries of a three-dimensional area with some given intensity value, separating arterial vessels, viens and bones. It is necessary to correctly determine the intensity threshold, limiting it from below to suppress noise and from above-to exclude surrogate structures from the image (Figure 2).

The process of reconstruction of the vessels after the surgery is more complicated, due to the need of creating an area that represents a flow-diverting device. In total, 3 approaches were considered for embedding such a region into the patient-specific geometry of the vessels with an aneurysm (mentioned above).

**Figure 3:**
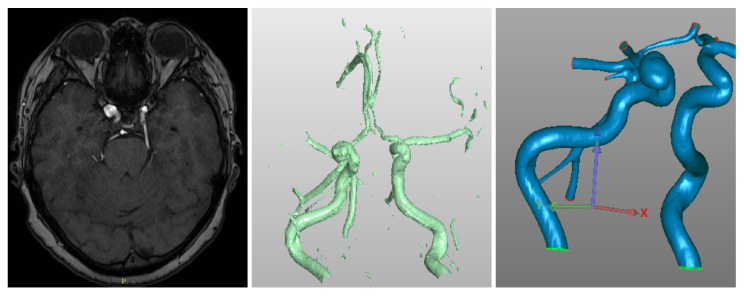
Overview of reconstruction process, from left to right: (**left**) a 3D model of vessels is built from a 3D CT-angio DICOM images; (**center**) Preparing continuos STL model via ITK Snap + 3D Viewer software; (**right**) finalization of the model via SolidWorks software (Boston, MA, USA).

**Figure 4:**
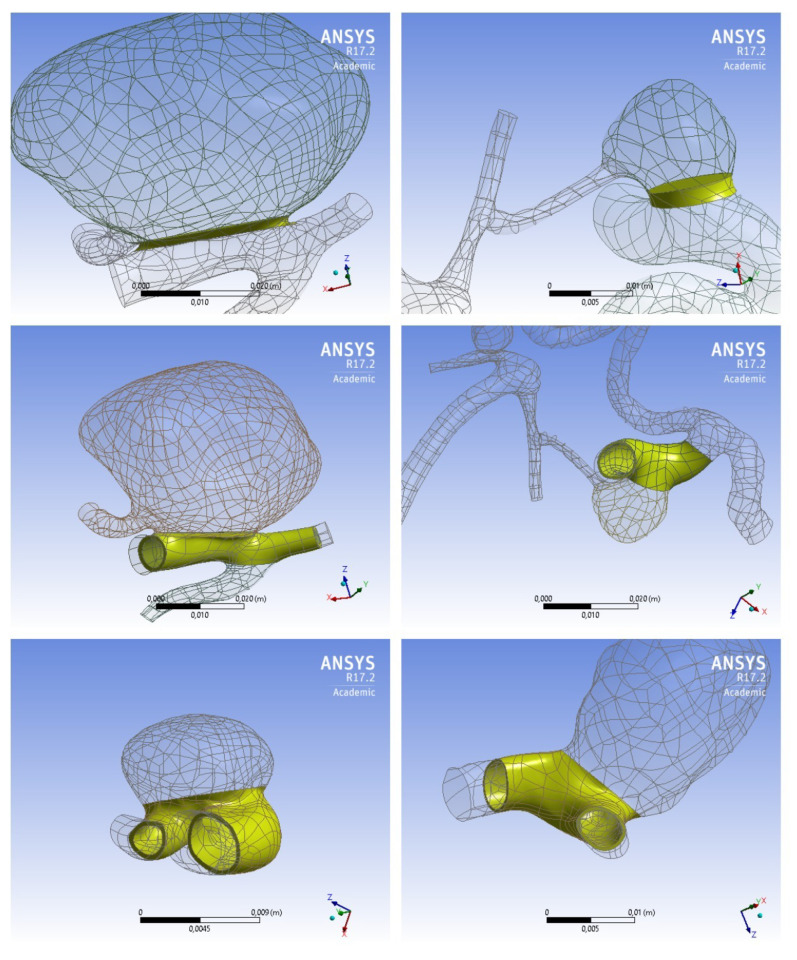
Approaches used to simulate virtual stent placement in cerebral vessels. Top row-scenario 1 like approach: patient 1 (on the **left**), patient 2 (on the **right**). The technique of the stent-layer between the aneurysm and the maternal vessel is performed as in [40]. Central row-the technique of the stent-tube located in the maternal vessel, performed as in [31] for patients 1 (on the **left**) and 2 (on the **right**), bottom row-the same stent-tube technique in patients 3 (on the **left**) and 4 (on the **right**).

The process of constructing three-dimensional geometry is shown in Figure 3. Examples of virtual stents of various structures are shown in Figure 4.

### 2.3. Numerical Calculations

Numerical calculations were carried out in the commercial software ANSYS 17.2 (license of LIH SB RAS) using the CFX solver. This solver uses a finite volume method centered on the [39] node. Previously scenario A was considered in [40], an insignificant dependence of the calculation results on the number of grid cells was shown. To solve numerical problem standart ANSYS solver was used, where Navier-Stokes equations were discretized (1):

(1) $$R^{t}\frac{\partial U}{\partial t}+\frac{\partial F_{1}}{\partial x_{1}}+\frac{\partial F_{2}}{\partial x_{2}}+\frac{\partial F_{3}}{\partial x_{3}}=0,$$

where $R^{-1}=\mathrm{diag}\left(\begin{array}{c} 0,1,1,1 \end{array}\right)$

(2) $$\begin{array}{c} U=\left(\begin{array}{c} p\\ u_{1}\\ u_{2}\\ u_{3} \end{array}\right),F_{1}=\left(\begin{array}{c} u_{1}\\ {u_{1}}^{2}+\hat{p}-\tau_{11}\\ u_{1}u_{2}-\tau_{12}\\ u_{1}u_{3}-\tau_{13} \end{array}\right),F_{2}=\left(\begin{array}{c} u_{2}\\ u_{1}u_{2}-\tau_{12}\\ {u_{2}}^{2}+\hat{p}-\tau_{22}\\ u_{1}u_{3}-\tau_{23} \end{array}\right),\\ F_{3}=\left(\begin{array}{c} u_{3}\\ u_{1}u_{3}-\tau_{13}\\ u_{2}u_{3}-\tau_{23}\\ {u_{3}}^{2}+\hat{p}-\tau_{33} \end{array}\right),\tau_{ij}=\mu \left(\frac{\partial u_{i}}{\partial x_{j}}+\frac{\partial u_{j}}{\partial x_{i}}\right), \end{array}$$

and $\hat{p}=p/\rho$, ρ=997 kg/m^3^—is the density of the liquid, *p*—is the pressure, *F*—is the external forces acting on the system, $\vec{u}=\left(u_{1},u_{2},u_{3}\right)$—are the components of the velocity vector, μ—is the dynamic viscosity of blood.

In the case of an unsteady flow of incompressible fluid derivatives with respect to the fictitious time $t^{\prime }$ are added to the system (1):

(3) $$\frac{\partial U}{\partial t^{\prime }}+R^{t}\frac{\partial U}{\partial t}+\frac{\partial F_{1}}{\partial x_{1}}+\frac{\partial F_{2}}{\partial x_{2}}+\frac{\partial F_{3}}{\partial x_{3}}=0,$$

where $R^{-1}=\mathrm{diag}\left(\begin{array}{c} 0,1,1,1 \end{array}\right)$.

It was previously shown that modeling the flow in the aneurysm zone using a non-Newtonian fluid flow regime is adequate from the physiological point of view [41]. In preliminary calculations, we did not reveal significant advantages of any of the models [42], therefore, having studied the literature, we decided to use Casson model of viscosity for aneurysm dome after stenting. In [40], a significant advantage of calculations was shown when using a mixed (in the sense of the Newtonian nature of the environment) viscosity setting: a more physiological description, a higher rate of convergence of the calculation. Using the second invariant of the strain rate tensor of an incompressible fluid: $\dot{\gamma }=\sqrt{\varepsilon_{ij}\varepsilon_{ij}}$ (4), we determined above mentioned model of viscosity:

(4) $$\mu ={\left(\sqrt{\tau_{0}/\dot{\gamma }}+\sqrt{\mu_{0}}\right)}^{2},$$

where $\tau_{0}=0.04$ dyne·s/cm^2^ is the yield point, $\varepsilon_{ij}$ is the component of the strain rate tensor, $\mu_{0}=0.04$ dyne·s/cm^2^ is the Newtonian viscosity of blood. It should be noted that $\mu_{0}$ and $\tau_{0}$ are constants corresponding to the empirical data on the properties of blood [43]. Examples of other non-Newtonian fluid models are provided in Appendix A.

The porous medium was modeled by adding a term (5) corresponding to external mass forces to the momentum Equation (1):

(5) $$F_{i}=-(\sum_{j=1}^{3}D_{ij}\mu u_{j}+\sum_{j=1}^{3}C_{ij}\frac{1}{2}\rho |u|u_{j}),$$

where $C_{ij}$, $D_{ij}$ are the given tensors. The convective term ($C_{ij}$) is assumed to be zero, because in the region of interest for the application of such a model, the velocities have small values, and we have the second order in terms of velocity in the expression. The dissipative term ($D_{ij}$) is set by the unit tensor in the absence of a preferred direction (scenarios A and B) and is determined according to an orthonormal basis, one normal of which is orthogonal to the stent-plug plane (which is built in scenario A) to set the viscosity in scenario C. Since the process of aneurysm occlusion during endovascular operations takes from several weeks to several months, in this case it was appropriate to consider a steady hydrodynamic setting, but to check the results, a non-steady setting was also considered. Both problems were solved without taking into account the effect of the interaction of blood flow with the stent and the artery wall (the problem with rigid walls).

To set the boundary conditions at the entrances of patient 1, we used the data of endovascular hemodynamic monitoring carried out using the Volcano ComboMap device at the Meshalkin Clinic Neurosurgery Department. The ComboWire sensor of the device, which has a diameter of 0.36 mm and a length of approximately 2 m, allows high accuracy to simultaneously measure pressure (piezoelectric method) and blood flow rate (ultrasonic Doppler method) with a radiation frequency of 12 MHz. The pressure values at the outlets were set by us in accordance with those measured for patient 1 and the averaged measurement data for patient 2. For patient 1, a patient-specific velocity graph was obtained [44,45]. To set the boundary conditions at the entrances in the circle of Willis for the patients 2, 3, 4 anonymized data of MRI monitoring of a large group of healthy patients [46]. Without loss of generality, we can use these data, since the aneurysm has a local effect on the hemodynamics of cerebral vessels. The no-slip condition was set on the walls.

In the non-stationary case, to determine the boundary conditions for all configurations, a velocity profile of patient 1 was taken and was placed uniformly at the entrance of the internal carotid artery. For all patients except patient 1 this profile was scaled (see how in Appendix D) with respect to the diameter of the vessels and fantom flowrate data from [46]. These values are also valid for the configuration with pathology, since the aneurysm makes changes in the blood flow locally, we may assume that proximally we have a conditions of a healthy vessel.

**Figure 5:**
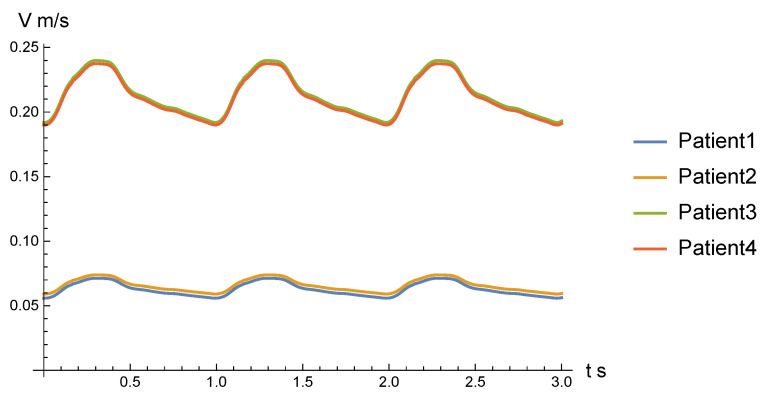
Velocity profiles applied at the inlets for all patients (patients 1,2-profiles for ICA, patients 3, 4-profiles for MCA). The volumetric distribution of blood flow in the cerebral vessels for scaling in relation to patients without intraoperative measurements was carried out on the data from [46].

At the outlets, opening-condition was set, but in further studies it is planned to construct a pressure profile near with the outlets, based on the average statistical intraoperative measurements carried out at the NMRC ac. Meshalkin [44]. The no-slip condition was set on the lumen wall. The opening condition was also set at each outlet, which, in the event of vortices appearing near them, allows the fluid to freely flow back into the configuration. The exact values of the velocity are presented in Table 2, and the graphs of the inlet velocity profiles are presented in Figure 5. Period of simulation was equal to 3 s and time step of 0.01 s has being considered. In total, the solver took 300 time steps for each case. To present the results, the following time points were chosen: $t_{1}$ = 2.03 s, $t_{2}$ = 2.25 s, $t_{3}$ = 2.36 s and $t_{4}$ = 2.70 s.

In a previos experience we faced with convergence effects during the 1st second of simulation, that is why to evaluate the results only the last second was considered.

### 2.4. Statistical Analysis

A comparison was made between the theoretical and experimental blood flow profiles. To identify the differences between the considered profiles, the Pearson test with 59 degrees of freedom was used. To analyze the results of numerical simulations first Shapiro-Wilks test was used to verify Gauss distribution afterwards Kolmogorov-Smirnov test was used [47].

## 3. Results

### 3.1. Steady Simulations

Validity of using of uniform velocity boundary conditions at inlets is of importance. To prove the establishment of the Poiseuille velocity profile with the vessel in the ANSYS CFX package, the planes of blood velocity distribution in the vessel were plotted at a distance of 0, 0.25 d, 0.5 d, 0.75 d and d from the inlet of the vessel configuration (d-corresponds to the vessel diameter at the inlet). A graph of exact Poiseil velocity profile was also plotted on a plane to discover differences between exact and numerical data obtained. The flow rate of an incompressible fluid with constant viscosity in a thin cylindrical tube of circular cross section under the influence of a constant pressure difference is determined by the formula [48]:

(6) $$v\left(r\right)=\frac{P_{1}-P_{2}}{4L\eta }*\left(R^{2}-r^{2}\right),$$

where *v*—fluid velocity, *r*—distance from the axis of the vessel, $P_{1}$–$P_{2}$—pressure drop at the inlet and outlet of the vessel, η is the dynamic viscosity coefficient, *R*—radius of the vessel, *L*—length of the vessel. $r_{i}$ of the same points that were used to build the previous graph were taken as *r* for plotting the graph.

**Figure 6:**
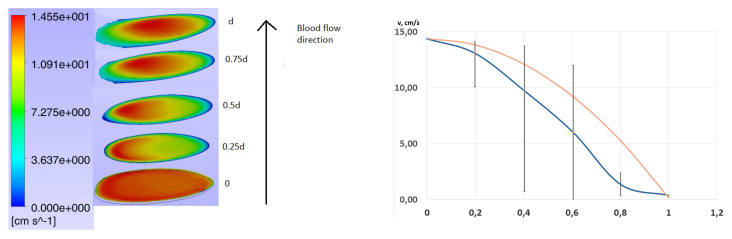
(On the **left**) Establishing of a Poiseuille velocity profile in the vessel with respect to distance from the inlet, where 0 corresponds to inlet boundary conditions, 0.25 d, 0.5 d, 0.75 d and d–slices, corresponds to value of the velocity through vessel slices, which are located at the correspond distance from the inlet (d-corresponds vessel diameter at the inlet), (on the **right**) Numerical (blue in the graph) and exact (orange in the graph) solutions for blood flow velocity profile in the vessel at a distance of d from the inlet.

According to the results of statistical analysis using the Pearson criterion with 60 degrees of freedom, the agreement between the numerical and exact velocity profiles was obtained with *p*-value = 0.986. Pearson’s test with 49 degrees of freedom was used for evaluation in the central zone of the vessel. As a result, the velocity profiles match with with *p*-value = 0.999. It is numerically proved that the Poiseuille profile is established in the vessel at a distance of 1 vessel diameter from the inlet with a high statistical accuracy. Thus we may conclude that monotonous profile is suitable for the simulations statistically well. The results are shown in Figure 6.

In our work, we assume that it is the change in *WSS* values that can affect the subsequent recanalization of the aneurysm. Please note that the combined use of Newtonian and non-Newtonian fluid models not only makes the problem more physiological, but also significantly increases the rate of convergence of the solution (up to 2 times, see Table 3)-which is expected due to the addition of one more dissipative term to the original Equation (1) compared with the case of constant viscosity for one of the flow regions.

**Figure 7:**
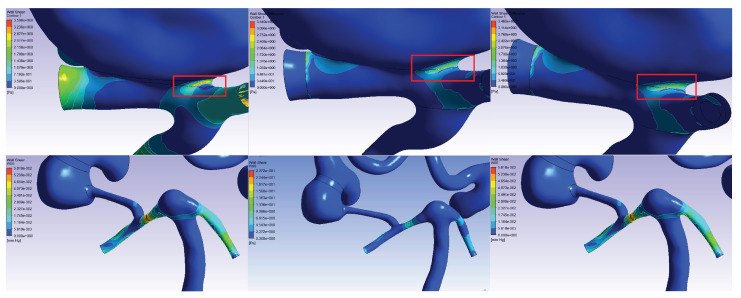
The Δ*WSS* (7) for 3 scenarios of steady numerical modeling: (on the **left**) scenario A, (in the **center**) scenario B, (on the **right**) scenario C. The first and second lines show the stress values for Patients 1 and 2, respectively.

**Figure 8:**
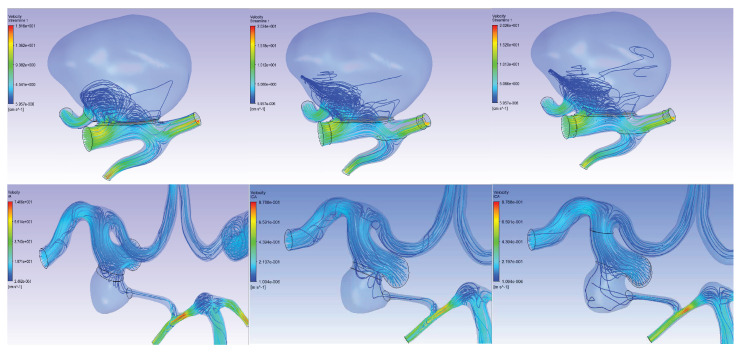
Velocity field streamlines for 3 scenarios of stationary numerical simulation; (on the **left**) scenario A, (in the **center**) scenario B, (on the **right**) scenario C. Streamlines for Patients 1 and 2 are presented on the first and second lines, respectively.

When analyzing the data of numerical calculations of stationary problem (Figure 7) for all three scenarios, we found a decrease in blood flow (Figure 8) in the area occupied by the cerebral aneurysm from the carotid artery basin, which was adequate to the clinical parameters. At the same time, when comparing the preoperative case (without stent) and the postoperative case (after stent deployment) in all three scenarios of patient-specific simulation, we noted an increase in the *WSS* values in the area of the aneurysm neck, (see Table 3) for patient 1, the absence of noticeable changes for this parameter for patient 2, and dramatically high increase for the patients No 3 and 4. Thus, The first conclusion is that an increase in Δ*WSS* values in the cervical region indicates aneurysm occlusion (corresponds to Patients 1, 3, 4) and small values of this quantity correspond to not occlusion (corresponds to Patient 2). However, we are also interested in the issue of adequate endothelialization of the stent in the postoperative period. It is well known [49] that the mechanism of endothelization is regulated by pulsatile shear force—that is why it is necessary to study non steady simulations for this problem.

Table 3 shows that comparing scenarios B vs. C the determination of the direction of energy dissipation by the blood flow during the passage through the porous area does not significantly affect the results of numerical simulation.

### 3.2. Unsteady Simulations

**Figure 9:**
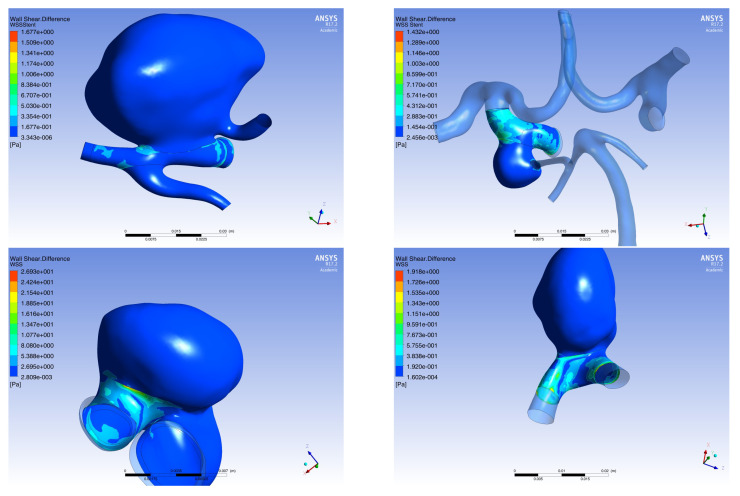
Distribution of Δ*WSS* at time point $t_{2}$: First line: (on the **left**) Patient 1, (on the **right**) Patient 2; Second line: (on the **left**) Patient 3, (on the **right**) Patient 4.

As a result of solving the non-stationary problem, the data of the stationary calculation were refined and the shear stresses on the walls (*WSS*) were obtained for all stent configurations in all 4 virtual patients (Figure 9). In the calculations, the main attention was paid to the value:

(7) $$\Delta WSS=WSS_{aftertheoperation}-WSS_{beforetheoperation}$$

in each cell of the calculation scheme. Data on the values of Δ*WSS* for each Patient are presented in Table 4. The distribution of Δ*WSS* in all patients, both in stationary and non-stationary cases, at all time points is presented in Appendix C.

It is known from the literature [50] that an increase in shear stress inhibits the proliferation of vascular endothelial cells in vitro, causing cell cycle arrest, and in vivo, significant proliferation of endothelial cells has been confirmed after a decrease in [51] shear stress.

The values presented in Table 4 show that the borderline between the normal case and the cases with complications is very transparent. Deviation of the maximum value of Δ*WSS* for the selected time points is about 20–30% higher or lower (for Patients 4 and 2-boundary patients in the cohorts) in comparing with values of Patient 1, whose stent is working properly, leads to either stenosis or no occlusion, respectively.

**Figure 10:**
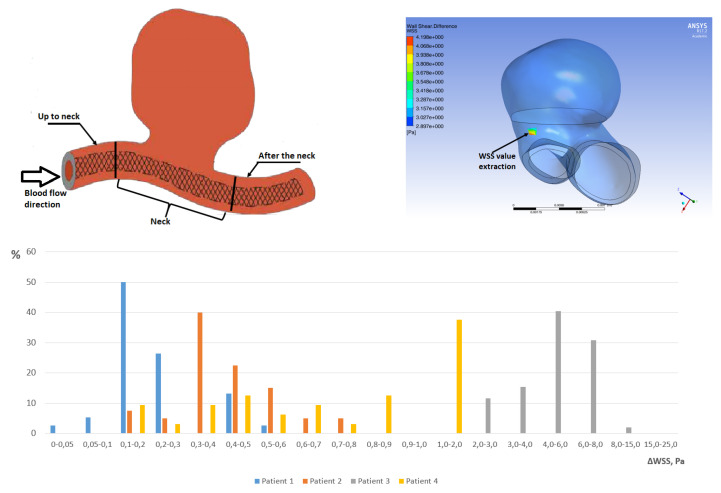
Analysis of Δ*WSS* distribution over all mesh cells, located at the stent surface in the “after the neck” compartment (as it is shown). Horizontal axis corresponds to Δ*WSS* values interval, vertical axis means the percent of mesh cells having Δ*WSS* value in the corresponding interval. In all cases the quantities was presented at the time point $t_{2}$ (Other time points were also analysed and no sufficient differences were found).

To confirm the obtained data (that we deal with real effects and not with data outliers), histograms of the Δ*WSS* distribution over the stent surface were constructed (Figure 10). Histograms confirmed all simulation results and provided another way of preoperative risk assessment by examining the statistics of the Δ*WSS* distribution over the stent surface. Histograms of the Δ*WSS* distribution for all patients at all time points $\left(t_{1},t_{2},t_{3},t_{4}\right)$ are presented in Appendix B. We also calculated such diagrams for other two regions (accordin to Figure 10) but no statistical differences between clinical outcomes were found.

(8) $$RSI=\frac{\underset{S}{\;\int \int \;}\tau \;dS}{S},$$

*S*—stent surface area, *τ*—*WSS*.

Shapiro-Wilk test showed Gauss distribution of *WSS* over the stent surface in Patients 2 and 3, and non Gauss distribution in Patients 1 and 4 (*p* < 0.05). The Kolmogorov-Smirnov criterion showed sufficient differences between Δ*WSS* distribution (*p* < 0.01) regarding to different cohorts of outcomes.

## 4. Discussion

### 4.1. General

The results confirmed all hypotheses. The stent was successfully modeled and installed in patient-specific vessel configurations. Please note that the goal of such modeling should always be to obtain a sufficient predictive model. That is such a model that will predict with good, from the point of view of statistics, accuracy a positive or negative outcome on the installation of a flow-redirecting device. It has been demonstrated that the method of stent modeling using a porous medium is well applicable in the problem of assessing the risk of aneurysm recanalization or arterial occlusion. The calculation results were presented in order to demonstrate the effectiveness of the used stent model, which allows us to reduce the computational load, in comparison with other models [52,53]. We believe that the latter approach is more adequate for the task of assessing the risk of arterial rupture by a flow-redirecting device, since in this case it is really important to understand the location of the threads in space, their curvature and torsion, which ultimately determine the amount of stress transmitted to the artery. The obtained results are consistent with the results obtained earlier [54], which confirms the correctness of our conclusions, however, the quantitative threshold between non-occlusion-norm and stenosis was revealed for the first time. The novelty of the approach we used is also in the understanding of RSI calculation. Due to a hugh variety of patient specific data we calculated RSI value for the difference between post and preoperative virtual surgery simulations. The data obtained are presented in the Table 5.

### 4.2. Boundary Conditions

Numerical proof of the establishment of the Poiseuille velocity profile in the vessel was performed for the first time for such simulation. It shows that the use of a profile of a complex structure is redundant and will not give any statistically significant advantages in the course of a numerical calculation. Figure 6 shows that the maximum deviations from the profile are formed near the vessel wall, which can be explained by two factors. First, in preliminary calculations, we have shown that the characteristics of the inflation zone, which must be specified in the numerical calculation of the flow of a viscous fluid, have a significant effect on the numerical solution. Secondly, the real boundary conditions on the wall have not been fully identified, and the choice of the sticking condition, although it is the gold standard in the field of solving such problems, is hardly 100% correct from the physiological point of view, which is undoubtedly a certain limitation of such studies. By the way, the profile obtained during the simulation is much more similar to the experimental one obtained in [55], than exact solution of Poiseuille, that is shown in the Figure 6. However, we note that the choice of other boundary conditions requires both a greater theoretical base in this area, which is almost absent, and a large number of laboratory and preclinical studies, which do not give correct response [56]. The joint use of Newtonian and non-Newtonian fluid models not only makes the problem more adequate from the physiological point of view, but also significantly increases the speed of solution convergence [40]. This approach is widespread in CFD for delimination of numerical artefacts in the solution [57]. We recommend other researchers to use a similar approach to increase the rate of convergence of blood flow modeling in an aneurysm excluded from the blood flow.

### 4.3. Flow-Divertion Device Design

As a result of numerical calculation, it was shown that when passing through a porous region simulating stent cells, the method of specifying the loss tensor (5) does not significantly affect the simulation results. This means that in order to simplify the formulation of a numerical experiment an isotropic tensor can be used and this will not detract from the quality of the numerical result obtained in this case.

The main quantitative hemodynamic parameter, which is studied in this work, is the magnitude of shear stresses (*WSS*). In the literature, a huge influence is attributed to the value of this parameter, which it has on the risk of aneurysm growth and rupture [50]. There are entire areas in computational hemodynamics that tend to have conflicting views about the risk of aneurysm rupture [58,59,60,61]. However, in the present work, we are rather speaking about the conditions of normal endothelialization of flow-redirecting devices, which is influenced by such factors as the clinical history of patients, the geometry of the flow area, and blood chemistry. All patients were seen at the same center by the same surgeon and were selected for this study precisely on the basis of the best match between the first (clinical) and last (chemical) factors. It is the geometry of the flow region that can be considered a significant difference under the conditions of endothelialization. Thus, by revealing the fact how exactly the hemodynamic characteristics of a particular patient affect endothelialization, we solve the problem of the influence of the geometry of the stent placement area on the possible development of postoperative complications of one kind or another in the future. At the same time, of course, clinical and chemical factors remain unexplored. Undoubtedly, the study of the influence of these factors will allow us to prevent the development of complications during the installation of flow-redirecting devices at a new level.

Surgeons want a universal tool to determine the risk of aneurysm rupture. This study is an important step towards the creation of this tool. However, everything depends on the speed of building the model and the speed of calculations. An attempt to automate the construction of patient-specific configurations stumbled upon a number of insurmountable questions: what part of the vessel can be removed to increase the calculation speed without increasing the error of the result, how to make the process of segmentation of DICOM images more robust, what, in the end, boundary conditions in the stent location area to apply (sticking or slippage and why) and what models of blood viscosity. A lot of progress has been made in this direction [62], however, for cerebral hemodynamics with adjacent arteries and veins, fully automatic algorithms still face difficulties, such as: ingrowth of vessels into indentations, the presence of spirals or clips, presence of shunts or foreign objects [63].

### 4.4. Limitations

One of the limitations of the study is the fact that at this stage, the border in clinical effects is described quantitatively, but the scale of difference is very blurred so far. However, as it can be seen from Figure 10, the difference in *WSS* values for the described cases proximal to the aneurysm neck zone is not an outlier, but corresponds to a large number of calculated stent cells (from 38 to 50% of cells in each case). To clarify the border of the norm a detailed consideration of a larger number of clinical cases is required.

In addition, a separate part of the medical community considers it necessary to take into account the interaction of the wall and blood flow in any case-the solution of FSI problems. However, it is shown that the use of the FSI technique is far from always justified, and sometimes it can lead to numerical artifacts that distort the physics of the solution [64]. In addition, to be completely honest, data on the strength properties of the vessel wall are required, at least within the framework of a uniaxial test, in order to determine the vessel elasticity model in the FSI problem. Even if this can be done non-invasively, using the promising LIF approach described by us in [65], then the entire process of preparing data and solving a problem can take up to several days when solving such a problem on a desctop computer and up to several hours when solving it on our own computing cluster, which is still time-expensive and not can be used in clinical routine.

Another limitation of this study is the decision not to consider the concomitant factors of the disease: smoking, the presence of systemic diseases [66,67] and hereditary diseases of the cardiovascular system. However, taking into account the fact that the sample is small, it does not allow assessing the influence of these parameters on the outcome of operations. Also the limiting factor of the study is the lack of modeling of the ongoing chemical processes [68] that leads to thrombus formation and subsequent occlusion of the cerebral aneurysm, since the occlusion process is influenced by both endothelialization of the stent and thrombus formation inside the dome of the aneurysm.

## 5. Conclusions

This study shows that a proper flow-diverting device placement leads not only to a decrease in blood flow to the aneurysm dome, but also to an increase in *WSS* values in the cervical area of the aneurysm, regardless of the geometric implementation of the stent. A relationship between quantitative differences in changes of shear stresses at the stent walls and different clinical outcomes was demostrated. This work is valuable for the purposes of preoperative modeling as a basis for identifying possible postoperative complications (stenosis, non-occlusion) when using flow-redirecting devices.

## Figures and Tables

**Table 1:** Patient sample characteristics and clinical outcomes.

	Patient 1	Patient 2	Patient 3	Patient 4
Sex	w	w	w	w
Age	73	65	64	58
Aneurysm location	M1 ICA	M1 ICA	ICA	ICA
Status after the treatment	Success	Occlusion did not occur	In stent stenosis	In stent stenosis

**Table 2:** Exact values of the inlet velocity for each patient.

Patients	$t_{1}$	$t_{2}$	$t_{3}$	$t_{4}$
Patient 1	0.057 m/s	0.071 m/s	0.071 m/s	0.06 m/s
Patient 2	0.06 m/s	0.074 m/s	0.074 m/s	0.063 m/s
Patient 3	0.196 m/s	0.239 m/s	0.239 m/s	0.204 m/s
Patient 4	0.191 m/s	0.235 m/s	0.237 m/s	0.202 m/s

**Table 3:** Comparison of the results of hydrodynamic calculations with different scenarios for modeling a FDD. Here n1=7, n2=5, n3=6, n4=8 are the relative number of iterations of the preoperative scenario for cases 1, 2, 3, and 4, respectively. The first line contains the values for Patient 1, the second-for Patient 2, the third-for Patient 3, the fourth-for Patient 4.

Modeling Scenarios	Initial Configuration without Stent	Scenario A Plug	Scenario B FDD-Tube (Non-Isotropic)	Scenario C, FDD-Tube (Isotropic)
Flow to the aneurysm	1.62	1.34	1.17	1.18
g/s	0.39	0.17	0.13	0.32
	4.1	3.6	3.12	3.13
	3.72	2.81	2.58	2.55
*WSS*	2.37	3.59	5.34	5.36
maximum value, Pa	0.28	0.38	0.38	0.94
	38.57	34.48	25.45	25.26
	5.23	2.97	2.13	2.7
Number of iterations	7,358,495	+200%, −8%	+200%, +33%	+200%, +33%
number of nodes	5,487,579	+700%, −1%	+200%, +53%	+200%, +53%
	6,366,709	+100%, +0%	+200%, +173%	+200%, +173%
	8,416,665	+200%, +0%	+200%, +115%	+200%, +115%

**Table 4:** Maximum values of Δ*WSS* (7) for the selected time points and the clinical effect obtained with this stent placement.

Patients	$t_{1}$	$t_{2}$	$t_{3}$	$t_{4}$	Clinical Outcome
Patient 3	19.02 Pa	26.93 Pa	26.62 Pa	20.01 Pa	In stent stenosis
Patient 4	1.421 Pa	1.918 Pa	1.899 Pa	1.491 Pa	In stent stenosis
Patient 1	1.141 Pa	1.677 Pa	1.660 Pa	1.226 Pa	Norm
Patient 2	0.997 Pa	1.432 Pa	1.426 Pa	1.067 Pa	No occlusion

**Table 5:** Time dependent value of RSI for different clinical outcomes.

Patients	Total Shear Force, N	Area, m^2^	RSI, Pa	Result
Patient 1	0.00006	0.00071	0.09	Norm
	0.00008	0.00071	0.109	
	0.00008	0.00071	0.113	
	0.00007	0.00071	0.098	
Patient 2	0.00009	0.00053	0.172	No occlusion
	0.00012	0.00053	0.234	
	0.00012	0.00053	0.23	
	0.00008	0.00053	0.158	
Patient 3	0.00019	0.00018	1.029	In stent stenosis
	0.00025	0.00018	1.385	
	0.00027	0.00018	1.466	
	0.00021	0.00018	1.146	
Patient 4	0.00012	0.00038	0.306	In stent stenosis
	0.00015	0.00038	0.407	
	0.00016	0.00038	0.419	
	0.00013	0.00038	0.332	

**Figure A1:**
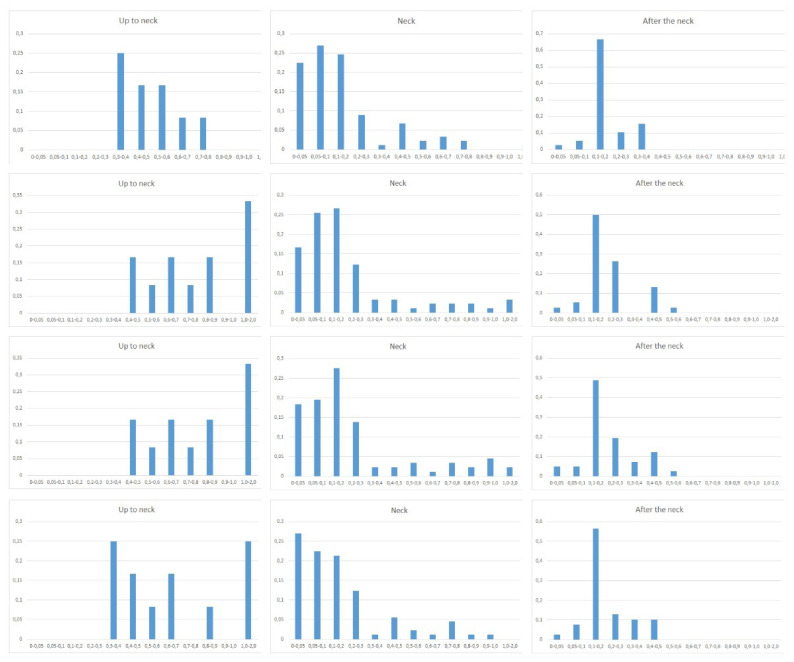
Histograms of Δ*WSS* distribution over the stent surface of Patient 1.

**Figure A2:**
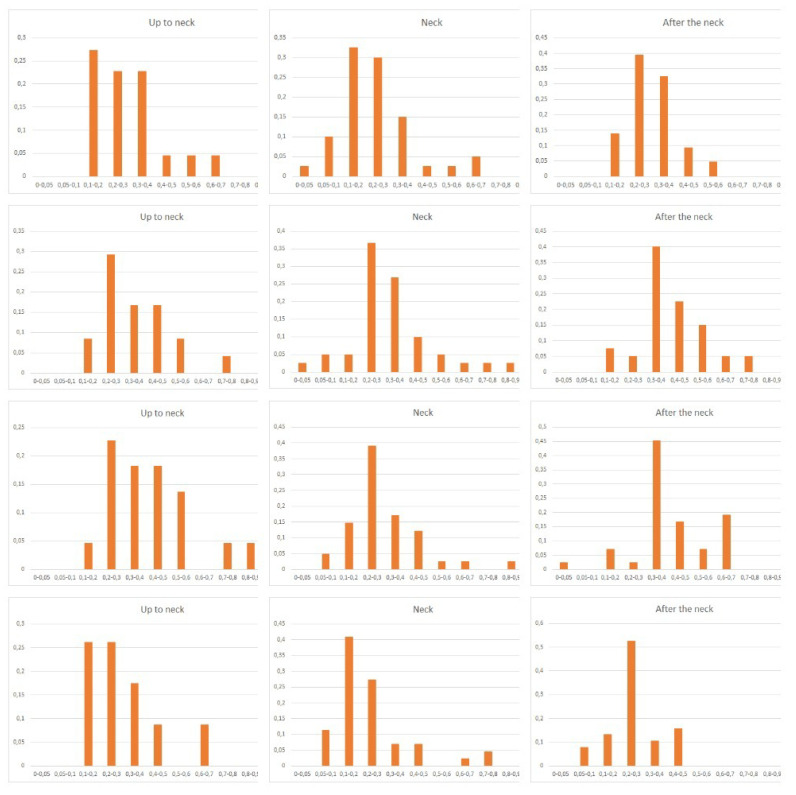
Histograms of Δ*WSS* distribution over the stent surface of Patient 2.

**Figure A3:**
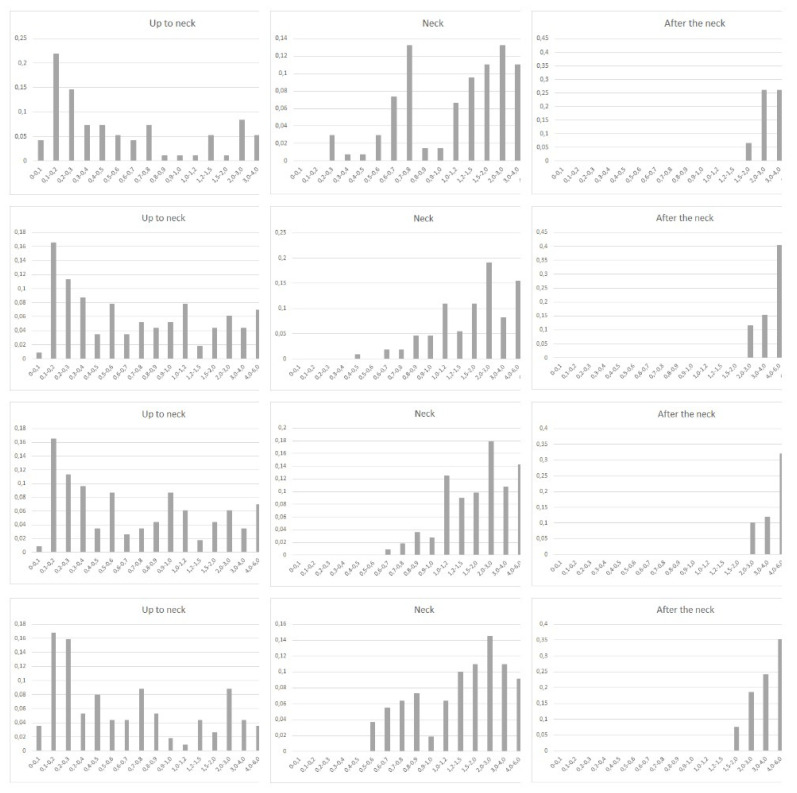
Histograms of Δ*WSS* distribution over the stent surface of Patient 3.

**Figure A4:**
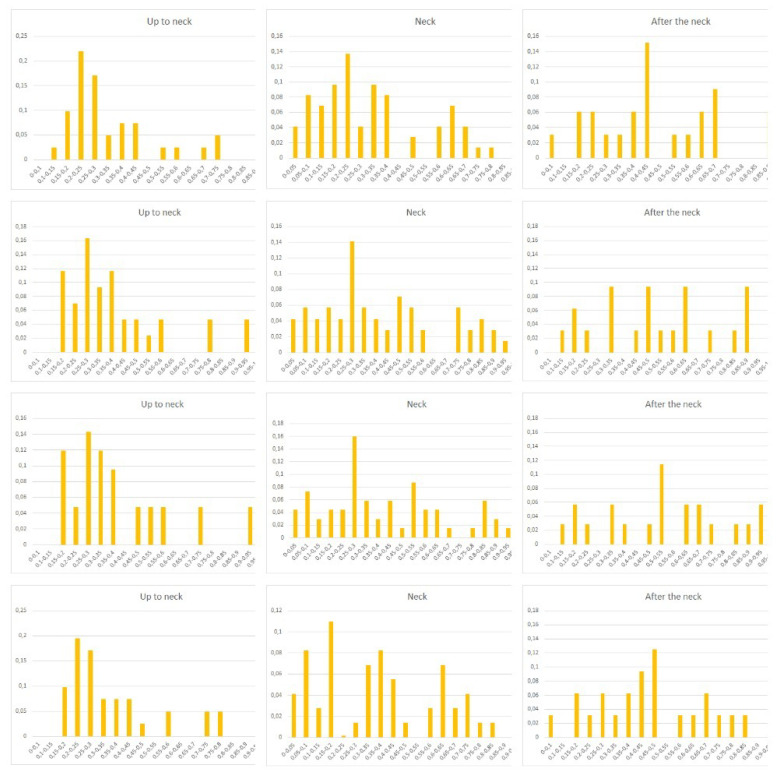
Histograms of Δ*WSS* distribution over the stent surface of Patient 4.

**Figure A5:**
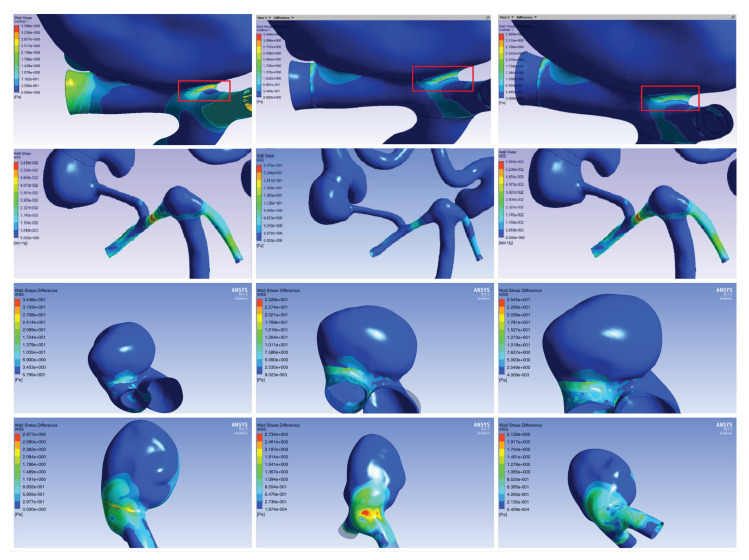
Distribution of Δ*WSS* for all patients (number of row corresponds to an identifier of patient) in steady simulations: Column 1: Scenario A, Column 2: Scenario B, Column 3: Scenario C.

**Figure A6:**
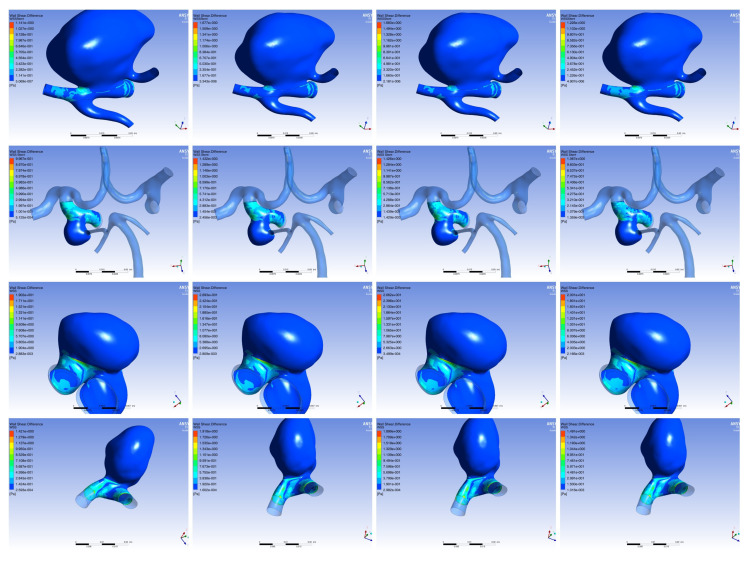
Distribution of Δ*WSS* for all patients (number of row corresponds to an identifier of patient) in a non-steady case for time points $t_{1}$–$t_{4}$: Column 1: $t_{1}$, Column 2: $t_{2}$, Column 3: $t_{3}$, Column 4: $t_{4}$. Only Scenario C of stent shape is presented.

**Figure A7:**
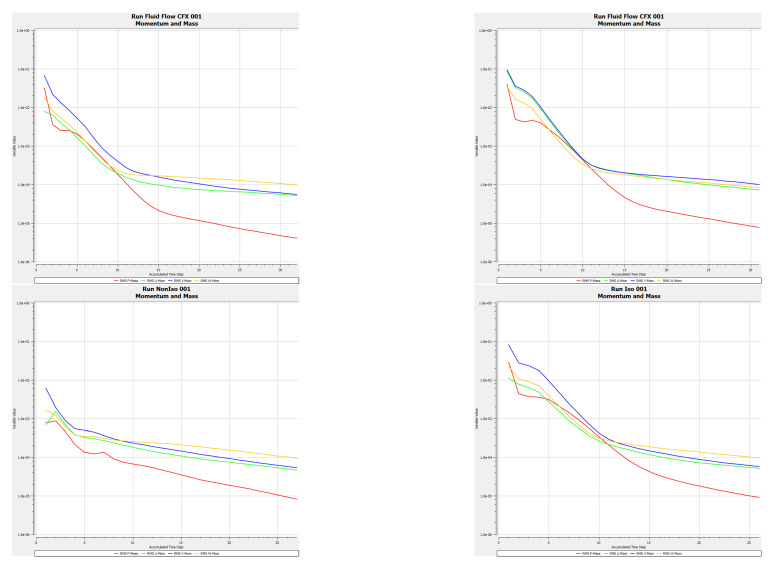
Residual convergence: first line (on the **left**) Without stent, (on the **right**) With plug, second line (on the **left**) With stent and non-isotropic tensor, (on the **right**) With stent and isotropic tensor.

## Data Availability

Details regarding the results of numerical calculations and statistical analysis are given in the appendices. Patient-specific geometries of the circle of Willis of all patients are in the Supplementary File of the article.

## Supplementary Materials

The following supporting information can be downloaded at: https://www.mdpi.com/article/10.3390/jpm12040652/s1, Patient 1–Patient 4.

## Abbreviations

The following abbreviations are used in this manuscript:

*WSS*	Wall shear stress
Max*WSS*	maximum value of wall shear stress in the region
FDD	flow diverter device
ICA	internal carotid artery
CT	computed tomography
MCA	middle cerebral artery
RSI	relative share index
CFD	Computational fluid dynamics
FSI	Fluid-structure interactions
LIF	Laser induced fluorescence

## Appendix A

It was previously shown that modeling the flow in the aneurysm zone using a non-Newtonian fluid flow regime is adequate from the point of view of the physiology of circulation processes. There are various models of non-Newtonian fluid that are used for the numerical analysis of hemodynamics, such as: *Herschel-Bulkley model.* In this model of a viscoplastic fluid, the viscosity *μ* is given by the following formula:

(A1) $$\mu =\frac{\tau_{y}}{\lambda \dot{y}}+K{\left(\lambda \dot{y}\right)}^{n-1},$$

where $\tau_{y}=0.0175$ Pa-yield point, K=8.9721 MPa·s-constant viscosity, λ=1 s-temporary constant, n=0.8601-power index;

Karo-Yasuda Model (A and B). The viscosity of this model is calculated as follows:

(A2) $$\mu =\mu_{\infty }+\frac{\mu_{0}-\mu_{\infty }}{{\left(1+{\left(\lambda \dot{y}\right)}^{a}\right)}^{(1-n)/a}},$$

where for model A: $\mu_{0}=0.056,\mu_{\infty }=0.00345$ Pa·s-viscosity at small and large shears, respectively, λ=1.902 s-temporary constant, n=0.22-power index, a=1.25-exhibitor Yasuda; for model B: $\mu_{0}=0.022,\mu_{\infty }=0.0022$ Pa·s-viscosity at small and large shears, respectively, λ=0.110 s-temporary constant, n=0.392-power index, a=0.644-exhibitor Yasuda.

## Appendix B

This section presents histograms of the Δ*WSS* distribution over the stent surface in all patients at all time points.

## Appendix C

This section presents the distribution of Δ*WSS* over the stent surface in all patients in stationary and non-stationary cases.

## Appendix D

(A3) $$Q=\frac{P_{1}-P_{2}}{X}$$

where *P*_1_–*P*_2_—pressure drop at the inlet and outlet of the vessel, $X=8L\eta /R^{4}\pi$ is the hydraulic resistance, *R*—radius of the vessel, *L*—length of the vessel, η is the dynamic viscosity coefficient. From here, according to Poiseuille’s law, the proportionality of velocity and volumetric flow follows Appendix D. In the non-stationary case, it is necessary to perform integration over the velocity over the period. Thus, since the integral is a linear functional, modifying the velocity profile allows one to modify the amount of volumetric blood flow. Since the actual velocity profiles are different for each patient, we used the velocity profile for one patient and thus the main contribution to the difference in the results obtained was made by the geometry of each individual patient and the shape of the stent.
